# Production of SnS_2_ Nanostructure as Improved Light-Assisted Electrochemical Water Splitting

**DOI:** 10.3390/nano9091244

**Published:** 2019-09-01

**Authors:** Haizeng Song, Han Wu, Yuan Gao, Ka Wang, Xin Su, Shancheng Yan, Yi Shi

**Affiliations:** 1 Collaborative Innovation Center of Advanced Microstructures, Nanjing University, Nanjing 210093, China; 2 School of Electronic Science and Engineering, Nanjing University, Nanjing 210093, China; 3 School of Geography and Biological Information, Nanjing University of Posts and Telecommunications, Nanjing 210023, China

**Keywords:** layered semiconductor, solution etching, SnS_2_ nanostructure, light-Assisted electrochemical water splitting

## Abstract

Tin disulfide (SnS_2_) has gained a lot of interest in the field of converting solar energy into chemical fuels in light-assisted electrochemical water splitting due to its visible-light band gap and high electronic mobility. However, further decreasing the recombination rate of electron-hole pairs and increasing the density of active states at the valence band edge of the photoelectrodes were a critical problem. Here, we were successful in fabricating the super-thin SnS_2_ nanostructure by a hydrothermal and solution etching method. The super-thin SnS_2_ nanostructure as a photo-electrocatalytic material exhibited low overpotential of 0.25 V at the current density of −10 mA·cm^−2^ and the potential remained basically unchanged after 1000 cycles in an H_2_SO_4_ electrolyte solution, which was better than that of the SnS_2_ nanosheet and SnS/SnS_2_ heterojunction nanosheet. These results show the potential application of super-thin SnS_2_ nanostructure in electrochemical/photo-electrocatalytic field.

## 1. Introduction

In recent years, there has been more and more research on the application of two-dimensional (2D) layered materials as field-effect-transistors (FETs) [1,2], photodetectors [3,4], photocatalysis [5], lithium-ion battery, etc. [6,7,8,9], which were dependent on the excellent electronic mobility and optical properties [10,11]. Besides, 2D layered materials have also been considered as a photocatalysis to water splitting in the next generation since its 2D conductive channel is beneficial to fast carrier transport and greatly reduce the recombination rate in the photoelectrode, and the larger surface area is conducive to rapid charge transfer and enhance electrochemical reaction at the interface, which could improve the reaction efficiency [12,13,14]. As an original member of semiconductor metal sulfides family, the hexagonal SnS_2_ is nontoxic, inexpensive, chemically stable in acidic and neutral solutions, and visible-light band gap of 2.2–2.4 eV [14,15,16]. In the last few years, it has been proved to be a promising photocatalyst in the application of dye degradation processes.

Recently, the SnS_2_ has been considered as a photo-electric-catalyst for water splitting. Sun et al. [12] first synthesized freestanding SnS_2_ single layers with three atom thickness by liquid exfoliation. The SnS_2_ single layers displayed excellent structural stability and increased density of states at the valence band edge, which achieved efficient visible light water splitting. Further, a series of regular hexagon-shaped SnS_2_ nanoplates were fabricated via a facile hydrothermal process by Fu et al. [16]. The SnS_2_ nanoplate-like products can efficiently delay photogenerated charge recombination, which showed good photocatalytic activity for H_2_ production. Moreover, Meng and co-workers [14] reported improved photoelectrochemical water splitting based on doped SnS_2_ nanosheet arrays with amorphization, vacancy, and gradient energy band through a hydrothermal method. Taking the above reports, SnS_2_ plays a key role in enhancing the visible light photocatalytic of water splitting. In this work, we reported the facile synthesis of super-thin SnS_2_ nanostructure via hydrothermal and solution etching method and applied it to light-assisted electrochemical water splitting. It is expected to exhibit better performance in light-assisted electrochemical water splitting.

## 2. Experimental Details

### 2.1. Preparation of SnS_2_ Nanostructure

All chemical reagents were of analytical grade and were used without further purification. SnCl_4_·5H_2_O, thiourea, and sulfur ammonia were purchased from Aladdin Industrial Corporation.

In the typical experiment, SnS_2_ nanostructure was synthesized by the solvothermal and etching solution methods. Briefly, 0.1753 g of SnCl_4_·5H_2_O and 0.0952 g of thiourea were dissolved in 30 mL of ethylene glycol by ultrasound to give a transparent solution. The mixture was then transferred into a Teflon-lined autoclave (50 mL) in an oven at 180 °C for 24 h. After cooling to room temperature, the precipitate was collected from the solution through centrifugal filtration, followed by washing several times using distilled water to remove the organic residues, and dried at 60 °C for 6 h. Next, the SnS/SnS_2_ heterojunctions were obtained by heat treatment at 500 °C for 30 min under Ar atmosphere. Finally, in order to obtain the SnS_2_ nanostructure by the etching solution method with sulfur ammonia for 20 min (10 and 30 min), the sample was washed several times using distilled water, and dried at 60 °C.

### 2.2. Materials Characterizations

Sample morphology was studied by a field-emission scanning electron microscope (FESEM; FE-SEM; JSM-7000F, JEOL Ltd., Tokyo, Japan). Transmission electron microscopy (TEM) and high-resolution transmission electron microscopy (HRTEM) images were obtained using a JEOL model JEM2100 instrument at an accelerating voltage of 200 kV (JEOL Ltd., Tokyo, Japan). The crystal phase properties of the samples were analyzed with a Bruker D8 Advance X-ray diffractometer (XRD) using Ni-filtered Cu Kα radiation at 40 kV, 40 mA and 2θ range from 10° to 60° with a scan rate of 0.1° per second (Bruker Daltonics Inc., Karlsruhe, Germany). Raman spectra were obtained using a Raman spectrometer (JY T64000) excited by the 488 nm line of an Ar^+^ laser under 22 mW (HORIBA, Ltd., Kyoto, Japan). Atomic force microscopy (AFM) images were taken by Cypher S microscopy (Oxford Instruments Asylum Research, California, USA). X-ray photoelectron spectroscopy (XPS) analysis (PHI5000 Versaprobe) was used to determine the chemical composition of the products (Ulvac-Phi Inc., Kanagawa, Japan).

### 2.3. Light-Assisted Electrochemical Water Splitting Measurements

Light-assisted electrocatalytic activity was measured at 25 °C in a three-electrode cell connected to a CHI-760E workstation (CH Instruments, Chenhua Co., Shanghai, China). SnS_2_ nanostructure (10 mg) (SnS_2_ and SnS/SnS_2_ heterojunction), acetylene carbon (1 mg), and 5 % polyvinylidene fluoride dimethylformamide solution (20 mg) were all mixed together. The obtained slurry was then coated onto carbon paper and then dried to form a thin-film electrode which was used as the working electrode. Meanwhile, Ag/AgCl (KCl filled) and a platinum wire both served as reference and counter electrodes, respectively. A 0.5 mol·L^−1^ H_2_SO_4_ solution was used as an electrolyte. The cell geometry is dual channel electrolytic cell. A 300 W Xe lamp served as a light source in the light-assisted electrochemical water splitting measurements. The lamp provided directional light with uniform intensity distribution and was filtered to simulate the solar spectrum before illuminating the sample. Polarization curves at a scan rate of 5 mV·s^−1^ were conducted in the above H_2_SO_4_ solution between 0 and −1 V. The light-assisted electrochemical water splitting performance of SnS_2_ nanosheet and SnS/SnS_2_ heterojunction were also measured by the same method.

## 3. Results and Discussion

SnS/SnS_2_ heterojunction could be synthesized through the hydrothermal and low-temperature annealing method. Then, the super-thin SnS_2_ nanostructure was obtained by the reaction between SnS/SnS_2_ heterojunction with ammonium persulfide. The conversion reactions:

SnS + (NH_4_)_2_S_2_→(NH_4_)_2_ SnS_3_ (1)

SnS_2_ + (NH_4_)_2_S→(NH_4_)_2_SnS_3_ (2)

(NH_4_)_2_S_2_→(NH_4_)_2_S + S (3)

**Figure 1:**
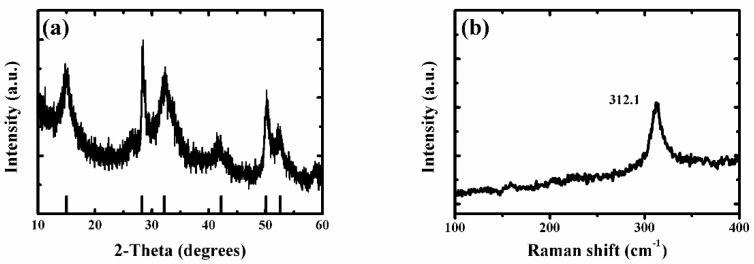
(**a**) The typical XRD pattern of the super-thin SnS_2_ nanostructure. (**b**) The typical Raman spectra of the super-thin SnS_2_ nanostructure.

**Figure A1:**
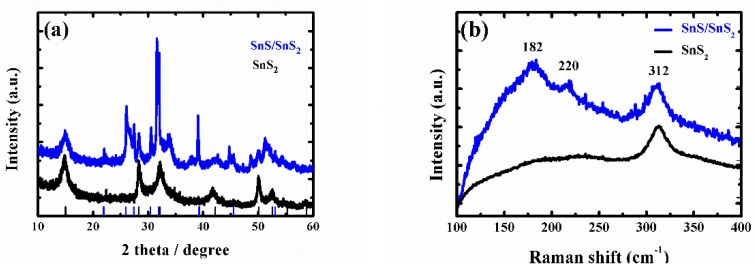
(**a**) XRD pattern of the SnS_2_ nanosheets and SnS/SnS_2_ heterojunction. (**b**) Raman spectra of the SnS_2_ nanosheets and SnS/SnS_2_ heterojunction.

As the ammonium persulfide solution reacted only with tin sulfide (from reaction (1)), the tin disulfide was retained. Because ammonium persulfide has poor stability at room temperature, some of the ammonium persulfides may be converted to ammonium sulfide (from reaction (3)) during the reaction. The tin sulfide will continue to react with ammonium sulfide (from reaction (2)). Therefore, samples with different morphologies can be obtained with different reaction times. From scanning electron microscopy (SEM), it was found that the mechanism was proved correct (see below). Therefore, the super-thin SnS_2_ nanostructure showed a thinner thickness and a smaller size and exhibited excellent light-assisted electrochemical water splitting performance. As shown in Figure A1a and Figure 1a, the X-ray diffraction (XRD) patterns of SnS_2_ nanosheet, SnS/SnS_2_ heterojunction nanosheet and super-thin SnS_2_ nanostructure samples were well crystallized. Moreover, all diffraction peaks of samples were very sharp, which indicated a high crystallinity. It was observed that diffraction peaks of the super-thin SnS_2_ nanostructure at 15.2, 28.2, 32.1, 41.9, 50.0, and 52.5° can be assigned to the (001), (100), (101), (102), (110), and (111) planes, respectively. Compared with SnS_2_ nanosheet and SnS/SnS_2_ heterojunction nanosheet (Figure A1a), we found that all the diffraction peaks of the super-thin SnS_2_ nanostructure were indexed to the hexagonal SnS_2_ (JCPDS No. 23-0677) [17,18,19]. In addition, it was found that there are no impurity peaks in the figures, indicating that the SnS_2_ material was obtained after solution etching. Furthermore, in order to demonstrate the sample was the super-thin SnS_2_ nanostructure after solution etching, the Raman analyzer was used to study the chemical structure (Figure A1b and Figure 1b). From the spectra in Figure A1b, the peaks of Raman spectra at 182, 220, and 312 cm^−1^ were SnS and SnS_2_, respectively. And Figure 1b shows the Raman spectra of the super-thin SnS_2_ nanostructure. The peak at 312.1 cm^−1^ was matched well with hexagonal SnS_2_ [20,21,22].

**Figure 2:**
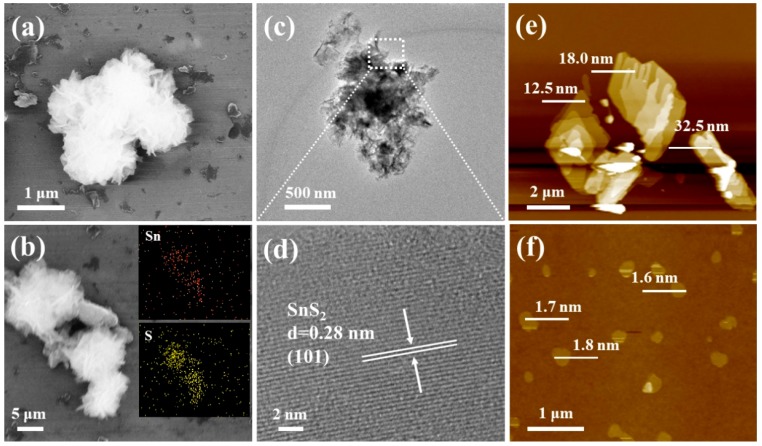
(**a**) The typical field-emission scanning electron microscope (FESEM) image of the super-thin SnS_2_ nanostructure. (**b**) The typical FESEM image and the energy-dispersive spectrometry (EDS) of the super-thin SnS_2_ nanostructure. (**c**) The typical transmission electron microscopy (TEM) image of the super-thin SnS_2_ nanostructure. (**d**) The typical high-resolution transmission electron microscopy (HRTEM) image of the super-thin SnS_2_ nanostructure, (**e**,**f**) atomic force microscopy (AFM) image of SnS_2_ nanosheet and super-thin SnS_2_ nanostructure.

**Figure A2:**
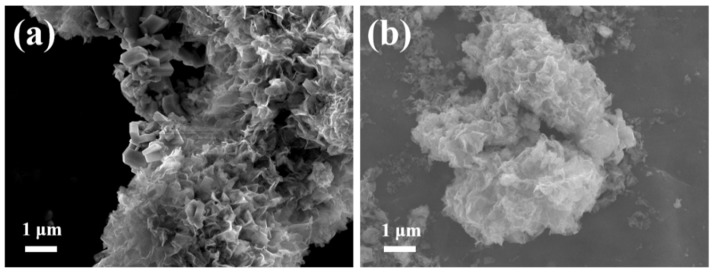
(**a**) The FESEM of the SnS_2_ nanosheets, (**b**) the FESEM of the SnS/SnS_2_ heterojunction.

**Figure A3:**
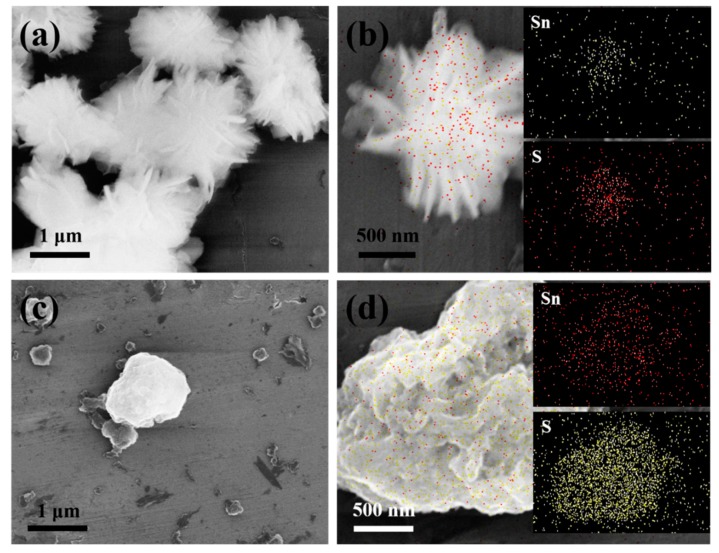
(**a**) The FESEM image of the SnS_2_ nanosheet with solution etching 10 min. (**b**) The FESEM image and EDS of the SnS_2_ nanosheet with solution etching 10 min. (**c**) The FESEM image of the SnS_2_ nanosheet with solution etching 30 min. (**d**) The FESEM image and EDS of the SnS_2_ nanosheet with solution etching 30 min.

Moreover, Figure 2, Figure A2 and Figure A3 show the field-emission scanning electron microscopy (HESEM) and transmission electron microscopy (TEM) images of the super-thin SnS2 nanostructure. The typical FESEM image of the super-thin SnS2 nanostructure displayed a typical super-thin nanosheet morphology. Moreover, in contrast with the FESEM of the SnS2 nanosheet and SnS/SnS2 heterojunction nanosheet in Figure A2, the super-thin SnS2 nanostructure showed the thinner nanostructure. This super-thin nanostructure could improve fast carrier transport and greatly reduce the recombination rate [12,23,24]. Figure 2b reveals the FESEM and energy-dispersive spectrometry (EDS) of the super-thin SnS_2_ nanostructure, the elemental mapping images confirm that the elements Sn and S were uniformly distributed among the sample [25,26]. Moreover, Figure A3 also shows the elements were uniformly distributed in the samples by the different time of solution etching. The uniform morphologies of the super-thin SnS_2_ nanostructure were also investigated by the TEM in Figure 2c,d. The super-thin SnS_2_ nanostructure with a lateral size of about 1 μm was observed. Additionally, the high-resolution transmission electron microscopy (HRTEM) image taken from the selected area in Figure 1c is shown in Figure 1d, which exhibited a crystal lattice spacing of 0.28 nm and belonged to the crystal facet (101) of hexagonal SnS_2_ [19,27,28]. Figure 2e,f shows the atomic force microscope (AFM) images of the SnS_2_ nanosheet and super-thin SnS_2_ nanostructure, which can further characterize the size and morphology. Figure 2f shows the super-thin SnS_2_ nanostructure with a thickness of 1.7 nm and lateral dimension of ~ 250 nm. Due to the above results, the super-thin SnS_2_ nanostructure showed a thinner nanostructure and a larger specific surface area than the SnS_2_ nanosheet. Therefore, the super-thin SnS_2_ nanostructure would exhibit excellent light-assisted electrochemical activity performance.

**Figure 3:**
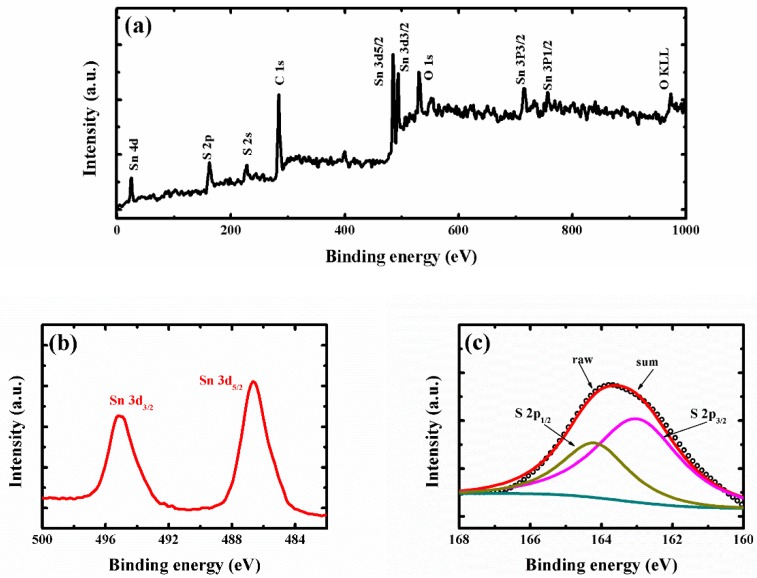
(**a**) Full XPS spectrum of the super-thin SnS_2_ nanostructure. (**b**) High-resolution XPS spectra of Sn 3d. (**c**) High resolution XPS spectra of S 2p.

To further confirm the surface-chemical states of the super-thin SnS_2_ nanostructure, the X-ray photoelectron spectroscopy (XPS) spectra were characterized in Figure 3. Figure 3a shows the wide scan spectrum of the super-thin SnS_2_ nanostructure, the Sn, S, C, and O elements were detected. It was indicated that the sample contained those elements and no other impurities could be found. The peaks at 495.2 and 486.6 eV were observed in Figure 3b, which was recognized as the 3d_3/2_ and 3d_5/2_ states of Sn^4+^ for the hexagonal SnS_2_ [8,14]. Moreover, the high-resolution XPS spectra for S 2p can be fitted with two peaks of 164.3 and 163.0 eV, corresponding to 2p_1/2_ and 2p_3/2_ states of S^2-^, respectively [29,30,31]. From the XRD, Raman, FESEM, TEM, and XPS data representation, the super-thin SnS_2_ nanostructure was successfully fabricated through the hydrothermal and solution etching method.

**Figure 4:**
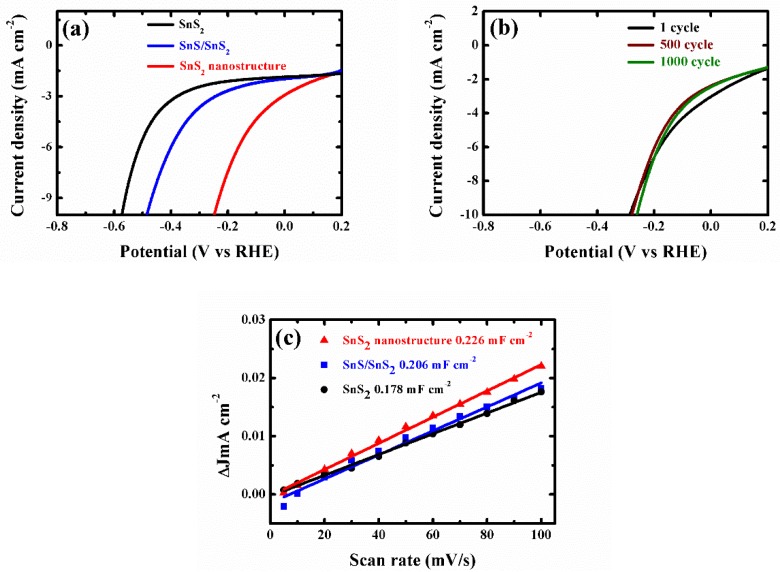
(**a**) Linear sweep voltammetry (LSV) curves of the SnS_2_ nanosheets, SnS/SnS_2_ heterojunction and super-thin SnS_2_ nanostructure for hydrogen evolution reaction (HER) in 0.5 M H_2_SO_4_ solution. (**b**) LSV curves of the super-thin SnS_2_ nanostructure before and after 1000 cycles. (**c**) The capacitive currents at −0.05 V as a function of scan rate for SnS_2_ nanosheets, SnS/SnS_2_ heterojunction and super-thin SnS_2_ nanostructure.

**Figure A4:**
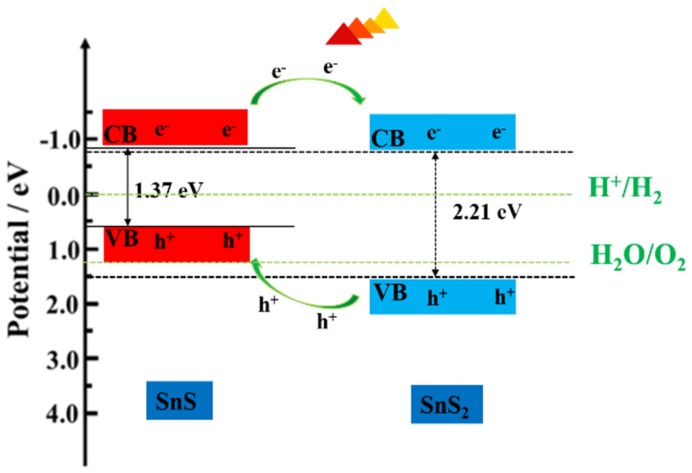
Schematic diagram for energy band matching and electron–hole separations.

**Figure A5:**
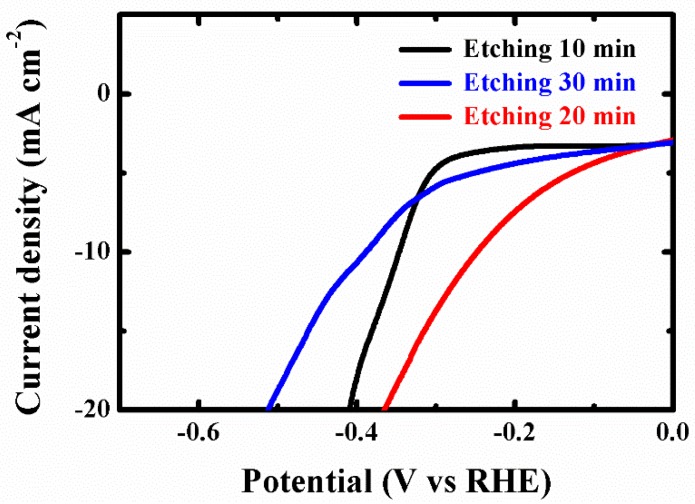
LSV curves of the SnS_2_ nanosheet by solution etching 10, 20, and 30 min with HER in 0.5 M H_2_SO_4_ solution.

**Figure A6:**
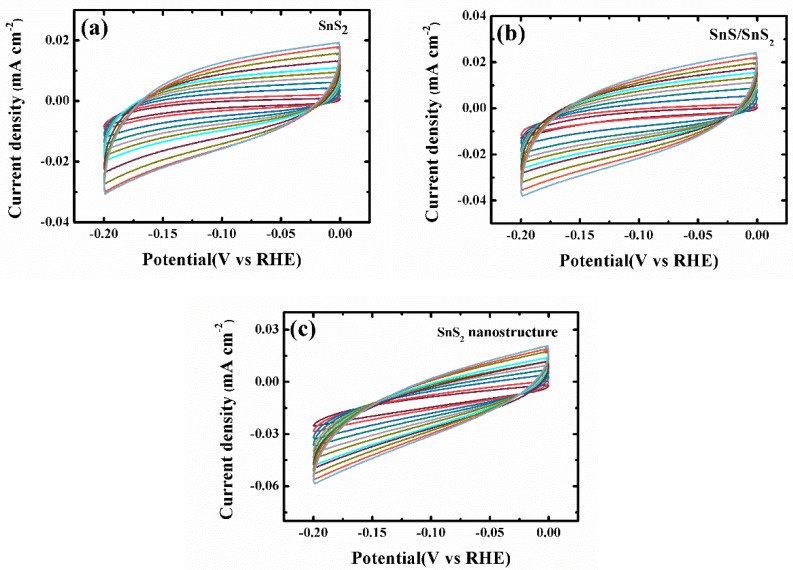
Cyclic voltammograms of (**a**) SnS_2_ nanosheets, (**b**) SnS/SnS_2_ heterojunction and (**c**) SnS_2_ nanostructure were measured in the non-faradaic capacitance current range at scan rates of 5, 10, 20, 30, 40, 50, 60 70, 80, 90, and 100 mV s^−1^.

**Figure A7:**
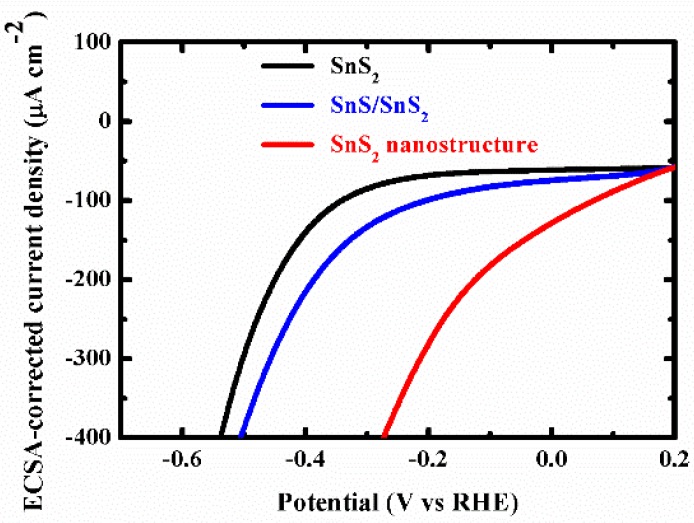
ECSA-corrected current densities versus applied potentials.

To evaluate the light-assisted electrochemical water splitting performance, the super-thin SnS_2_ nanostructure, SnS_2_ nanosheet, and SnS/SnS_2_ heterojunction sheet were measured by the typical three-electrode cell connected with a CHI 670E configuration in 0.5 M H_2_SO_4_ solution under a 300 W Xe lamp at the room temperature [14,16,32,33,34,35]. Figure 4a shows the linear sweep voltammetry (LSV) curves of the samples. The SnS_2_ nanosheet displayed poorer photocatalytic activity than the SnS/SnS_2_ heterojunction sheet. SnS/SnS_2_ heterojunction sheet showed improved light-assisted electrochemical water splitting performance, which depended on its specific energy band structure (Figure A4 shows that the photo-generated electrons on the CB of SnS can easily flow to the CB of SnS_2_ through the interface. In the same way, the holes on the VB of SnS_2_ are more positive than those of SnS and can be transferred to the VB of SnS and the VB edge level of SnS_2_. It can be efficient electron-hole pair separation and enhance photocatalytic activity). However, the super-thin SnS_2_ nanostructure displayed an overpotential of 0.25 V at the current density of −10 mA cm^−2^, which exhibited better overpotential than the SnS_2_ nanosheet and SnS/SnS_2_ heterojunction sheet and proved its superior hydrogen evolution reaction (HER) activity by the higher electrochemically active surface areas (Figure 4c and Figure A7). Meanwhile, to check the durability of the super-thin SnS_2_ nanostructure, the basically unchanged LSV curves can be observed from comparing before and after 1000 cycles. Additionally, as the as-prepared SnS_2_ nanostructures were further compared with different times of solution etching, the samples were also analyzed by the LSV curves under the same conditions in Figure A5. The sample with 20 min solution etching (super-thin SnS_2_ nanostructure) displayed the best HER performance of all samples (solution etching 10 and 30 min). Therefore, the electrochemically active surface areas (ECSA) of the super-thin SnS_2_ nanostructure, SnS_2_ nanosheet and SnS/SnS_2_ heterojunction sheet were measured by the double-layer capacitances (C_dl_). The C_dl_ was estimated through the investigated cycling voltammetry (CV) curves at different scan rates under non-faradaic region (Figure A6). As shown in Figure 4c, the super-thin SnS_2_ nanostructure exhibited larger C_dl_ value than that of SnS_2_ nanosheet and SnS/SnS_2_ heterojunction sheet. Interestingly, the super-thin SnS_2_ nanostructure exhibited larger ECSA than SnS_2_ nanosheet, which was regarded as higher catalytic activity. Moreover, better intrinsic catalytic activity for super-thin SnS_2_ nanostructure was further proved by its ECSA-corrected current densities in comparison with SnS_2_ nanosheet and SnS/SnS_2_ heterojunction in Figure A7 (detailed discussion in Appendix A). Due to the above results, the super-thin SnS_2_ nanostructure has a higher active surface area and more active sites, which can enhance HER property. 

## 4. Conclusions

In summary, the super-thin SnS_2_ nanostructure has been successfully synthesized through a hydrothermal and solution etching route. The super-thin SnS_2_ nanostructure offered excellent light-assisted electrochemical water splitting performance due to effectively capturing visible light, enhancing carrier density, rapid charge transfer and fast chemical reaction. Given these unique benefits, we believe that super-thin SnS_2_ nanostructure built on the other trend can provide a new potential application for electrochemical/photo-electrocatalytic devices.

## Appendix A

The SnS/SnS_2_ heterojunction nanosheet acts as a photocathode, the catalytic mechanisms may be responsible for the following reasons: As shown in Figure A5, when SnS_2_ is in contact with SnS to form p-n heterostructure, these photo-generated electrons on the CB of SnS can easily flow to the CB of SnS_2_ through the interface. It is considered that the CB position of SnS is more negative than the CB position of SnS_2_. In the same way, the holes on the VB of SnS_2_ tend to transfer to the VB of SnS and the VB edge level of SnS_2_ is more positive than that of SnS, which can cause efficient electron-hole pair separation and thus lead to enhanced photocatalytic activity.

To measure electrochemical double-layer capacitance (Cdl), the potential was swept nine times at each scan rate (5,10, 20, 30, 40, 50, 60, 70, 80, 90, and 100 mV/s) in the scan range from −0.20 to 0 V vs. RHE. Capacitive currents were measured in a potential range where no faradic processes were observed. The measured capacitive currents difference (∆J) at −0.05 V vs. RHE was plotted against scan rate and specific capacitance was determined from the slope of the linear fitting. The Cdl values for SnS_2_ nanosheet and SnS/SnS_2_ heterojunction sheet and super-thin SnS_2_ nanostructure are calculated to be 0.178, 0.206, and 0.226 mF·cm^−2^, respectively. The specific capacitance is converted into an electrochemical surface area (ECSA) using the specific capacitance value for a flat standard with 1 cm^2^ of real surface area. We use the specific capacitance (20–60 μF cm^−2^) of 40 μF cm^−2^ here to calculate the ECSA (Nature Communications, 2018, 9, 2452. and Nature Communications, 2019, 10, 399.) according to Equation (A1):

(A1) $$\mathrm{ECSA}=\frac{C_{dl}}{40\;\mu F/cm^{2}}cm_{ECSA}^{2}$$

**Calculated electrochemical active surface area:**

SnS_2_ sheet:

(A2) $$A_{ECSA}^{SnS_{2}}=\frac{0.178mF\;cm^{-2}}{40\;\mu F\;cm^{-2}\;per\;cm_{ECSA}^{2}\;}=4.45\;cm_{ECSA}^{2}$$

SnS/SnS_2_ heterojunction sheet:

(A3) $$A_{ECSA}^{SnS/SnS_{2}}=\frac{0.206mF\;cm^{-2}}{40\;\mu F\;cm^{-2}\;per\;cm_{ECSA}^{2}}=5.15\;cm_{ECSA}^{2}$$

Super-thin SnS_2_ nanostructure:

(A4) $$A_{ECSA}^{S-SnS_{2}}=\frac{0.226mF\;cm^{-2}}{40\;\mu F\;cm^{-2}\;per\;cm_{ECSA}^{2}}=5.65\;cm_{ECSA}^{2}$$
